# Seroprevalence and risk factors associated with *Pseudorabies virus* infection in Tibetan pigs in Tibet

**DOI:** 10.1186/s12917-018-1347-x

**Published:** 2018-01-22

**Authors:** Qingxia Wu, Hui Zhang, Hailong Dong, Khalid Mehmood, Zhenyu Chang, Kun Li, Suozhu Liu, Mujeeb Ur Rehman, Fazul Nabi, Muhammad Tariq Javed, Hongyun Zhu, Jiakui Li

**Affiliations:** 1 Key laboratory of clinical veterinary medicine in Tibet, Tibet Agriculture and Animal Husbandry College, Linzhi, 860000 Tibet People’s Republic of China; 2 College of Veterinary Medicine, Huazhong Agricultural University, Wuhan, 430070 People’s Republic of China; 3 University College of Veterinary & Animal Sciences, the Islamia University of Bahawalpur, Bahawalpur, 63100 Pakistan; 4 Departmet of Pathology, University of Agriculture, Faisalabad, Pakistan

**Keywords:** Pseudorabies (PR), Seroprevalence, Risk factors, Tibetan pigs, Tibet

## Abstract

**Background:**

Pseudorabies (PR) is an important emerging infectious disease that is characterized by fever, extreme itching and encephalomyelitis. However, it is still unclear whether Tibetan pigs are exposed to Pseudorabies virus (PRV) or not.

The present study was conducted to investigate the seroprevalence of PRV infection in Tibetan pigs in Nyingchi area of Tibet through enzyme-linked immunosorbent assay (ELISA). A total of 368 serum samples from Tibetan pigs were collected during 2015.

**Results:**

Results showed that 58 (15.76%) samples were found positive for PRV antibodies with further distribution of 18.23%, 13.42% and 6.25% from Nyingchi, Mainling and Gongbo’gyamda areas on the Tibetan plateau, respectively; along with 12.10%, 17.71% and 17.57% prevalence of PRV in juveniles, sub-adults and adults, respectively. The prevalence of PRV infection between male (14.61%) and female (16.84%) showed non-significant difference (*P* > 0.05). The risk factors of infection were found to be associated with feed type, age and altitude.

**Conclusions:**

The present study depicts a serious concern with a new emerging infectious disease in Tibetan pigs in Tibet, China.

## Background

Pseudorabies (PR) or Aujeszky’s disease is one of the highly contagious diseases, which is responsible for devastating outbreaks and considerable economic losses to the swine industry in China. It is caused by Pseudorabies virus (PRV), which is the member of genus Varicellovirus, belonging to subfamily Alphaherpesvirinae, and family Herpesviridae [1–3]. The domestic and feral pigs are the main natural hosts of this disease [4]. The PR is usually characterized by reproductive disorders such as miscarriage, fetal death and mummified fetus. The mortality rate of newborn piglets and abortion are up to 100% in some cases [5]. The infection in growing pigs usually leads to decrease in semen quality, and even death in some cases. Pseudorabies is highly hazardous and widely distributed contagious disease, and world health organization (OIE) has listed PR as class B animal disease, and listed as the secondary kind of animal disease in China [6].

The PR is an endemic and widespread swine disease in China from the last 5 years, and it is planned as one of the priority swine diseases for control [7, 8]. Recently, with the use of PRV vaccines, this situation has been controlled on large-scale pig farms [2]; however, the prevention and control of PR on small pig farms and free-ranging pigs is still a big challenge [9]. The regional elimination and eradication program of PRV is required in China as no region is declared free from PRV yet [6].

In China, PRV was first reported in cats in 1947, and subsequently found in swine [7]. Tibetan pig is relatively ancient in origin, indigenous and rare plateau type pig breed in the world and is the only high-altitude pasture pig breed in China [10, 11]. It is distributed in the Qinghai-Tibetan Plateau in China (Qinghai, Sichuan, Yunnan and eastern Tibet region). The seroprevalence of PRV infection in pigs in Qinghai, Sichuan and Yunnan areas is reported in local Chinese journals but hardly accessible to international readers, and that is summarized in Table 1.

**Table 1:** Prevalence of Aujeszky’s disease virus infection in pigs and in Tibetan pig distribution province, China

Province	No. tested	Test method	Positive (%)	Reference
Qinghai	456	ELISA	4.80%	Xiaoying Qi et al. 2015 (in Chinese)
Sichuan	1110	ELISA	17.61%	Kui Nie et al. 2013 (in Chinese)
Yunnan	1164	ELISA	35.40%	Xianghua Shu et al. 2010 (in Chinese)
Tibet	368	ELISA	15.76%	In present study

To date, no survey has been conducted to elucidate PRV infection and risk factors on PR in Tibetan pigs. The current study is conducted for the first time on seroprevalence of PRV infection in Tibetan pigs and it provides a reference basis for the PRV research in the future.

## Methods

### The study sites

**Fig. 1:**
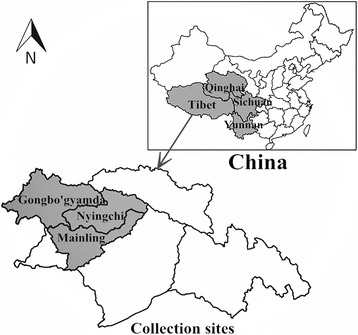
Geographic location of Aujeszky’s disease virus in four provinces, China. Map was created by using Mapinfo software; data are from the National Fundamental Geographic System website (www.dituhui.com)

The present study was carried out in Nyingchi Prefecture in southeastern Tibet, China. This area is geographically isolated from Tibet and Sichuan Provinces by Himalayan Mountains, and shares its borders with Yunnan and Qinghai Provinces [10–12]. Nepal is located on the southwest, while India and Myanmar in the south of the Nyingchi. The average elevation of the surveyed area is more than 3100 m above the sea level (Fig. 1).

### Samples

A total of 368 blood samples were collected from different slaughterhouses of three counties (Nyingchi, Mainling and Gongbo’gyamda) in Tibet during 2015 (Fig. 1). All samples were collected from non-vaccinated pigs. The blood samples were collected by anterior vena cava of pigs. After collection of blood samples, each sample was centrifuged at 3000×g for 20 min to separate the serum which was subsequently stored at − 20 °C. These samples were transported in ice bag to Huazhong Agricultural University for further analysis.

The serum samples were used for detection of IgG antibodies for PRV by a commercially available ELISA kit (Wuhan Keqian Biology Co., Ltd). All the protocols were followed according to the manufacturer’s instructions and the results were interpreted accordingly.

The results were based on the critical value (cut off) according to the formula: Critical Value = the average of Negative control OD_630_ + 0.15. The validity was ensured as: the average of Negative control OD_630_ - Positive control OD_630_ was ≥0.4. The samples were interpreted as negative: if sample OD_630_/ Negative controlOD_630_ was > 0.7 (cut off). The samples were interpreted as positive if sample OD_630_/ Negative control OD_630_ was ≤0.7 (cut off).

### Statistical analysis

The data were analyzed by the chi-square test using the procedure of SAS (Statistical Analysis System, Version 8.0). Statistically significant levels within factors and interactions were recognized, when probability (*P*) value was found *P* < 0.05. Odds-ratios were determined for the risk factors associated PRV along with 95% confidence intervals.

## Results

### Seroprevalence of PRV

A total of 35 (16.36%) out of 214 serum samples were found positive for antibodies to PRV in large-scale Tibetan pig farms, with the further distribution of 23 (20.00%), 11 (12.64%) and 1 (8.33%) from Nyingchi, Mainling and Gongbo’gyamda areas, respectively. Antibodies against PRV were detected in 23 (14.94%) of 154 serum samples by ELISA in free-ranging Tibetan pigs, with the further distribution of 14 (15.91%), 9 (14.52%) and 0 from Nyingchi, Mainling and Gongbo’gyamda areas, respectively. The results were statistically significant between large-scale and free-ranging Tibetan pigs (*P* < 0.05), as well as among different regions (Table 2).

**Table 2:** Descriptive characteristics and estimates of Aujeszky’s disease virus seroprevalence at large-scale Tibetan pigs and free-range Tibetan pigs

Variable	Large-scale Tibetan pigs		Free-ranging Tibetan pigs		Total	
	Samples	NO. Positive (%)	Samples	NO. Positive (%)	Samples	NO. Positive (%)
Nyingchi	115	23(20.00%)	88	14(15.91%)	203	37(18.23%)
Mainling	87	11(12.64%)	62	9 (14.52%)	149	20(13.42%)
Gongbo’gyamda	12	1 (8.33%)	4	0	16	1(6.25%)

The results of this study showed that a much higher proportion of seropositive were sub-adults (17.71%), followed by adults (17.57%) and juveniles (12.10%). There was non-significant difference between the sub-adults or adults and juveniles as shown in Table 3. The results revealed that the prevalence in female and male Tibetan pigs’ was 14.61% and 16.84%, respectively. The seroprevalence was 21.43%, 15.70% and 11.11% at the altitude of 2800 m, 3200 m and 3700 m, respectively. Statistical analysis revealed that the prevalence of PRV was significantly different (*P* < 0.05) at different altitudes.

**Table 3:** Association between Tibetan pig characteristics and Aujeszky’s disease virus serological status with corresponding chi square (χ^2^), *p*-value, odds ratio (OR), and confidence interval (CI)

Characteristic	No. tested	Positive		95% CI	MH Chi-sq *P* value	OR/reciprocal
		N	%			
Age						
Juveniles	124	15	12.10	6.9–19.2	*P* = 0.229	–
Sub-adults	96	17	17.71	10.7–26.8		–
Adults	148	26	17.57	11.8–24.7		–
Sex						
Male	178	26	14.61	9.8–20.7	–	0.84/ 1.18
Female	190	32	16.84	11.8–22.9	–	
Feed type						
Free-ranging	154	23	14.94	9.7–21.6	–	0.90 / 1.11
Large-scale	214	35	16.36	11.7–22.0	–	
Altitude						
3700 m	135	15	11.11	6.4–17.7	*P* = 0.027	–
3200 m	121	19	15.70	9.7–23.4		–
2800 m	112	24	21.43	14.2–30.2		–

### Risk factors for seropositivity of PRV

We evaluated four pre-identified probable risk factors including age, sex, feed type and altitude. Our results showed that feed type, altitude and age were significantly associated with an increase risk of PRV seropositivity in Tibetan pigs (Table 3).

## Discussion

Pseudorabies virus has been reported in many countries and it was first reported in the 19th century in the United States [7, 13, 14]. Infectious diseases have been serious threat for animal health and productivity in developing countries [15–19]. China is the world’s largest producer of swine, and PRV has become a major infectious disease threat to pig industry and causes serious issue. The lack of awareness and control measures against common infectious disease is a major cause of PRV in population. Although, the vaccination program was applied on many pig farms, but there were often occurrence and epidemic of this disease. The previous reports showed that the prevalence of PRV infection in pigs in Qinghai (4.80%), Sichuan (17.61%) and Yunnan (35.40%) provinces were very serious [5, 20, 21]. Tibetan pigs are mainly distributed in Qinghai, Sichuan, Yunnan and the eastern Tibet region. There are no previous published data regarding the prevalence of PRV infection in pigs. To the best of our knowledge, the present study provides the first report of PRV infection in Tibetan pigs.

In our study, the overall PRV seroprevalence was 15.76% in Tibetan pigs in Tibet, which was lower than that reported in pigs in Shandong (56.7%), Henan (53.7%), Beijing (24.0%), Tianjing (17.1%), Hebei (16.9%) and Liaoning (16.8%) [1, 8, 22, 23]. The low seropositivity of PRV in Tibetan pigs may be due to the evolution difference and unique characteristics of the plateau environment. On the other hand, Tibet’s unique mountains and rivers build a natural barrier to prevent or minimize the chances of spread of PRV.

Our results showed PRV infection among the different age groups of Tibetan pigs, a higher prevalence was detected in sub-adult (17.71%) or adult (17.57%) pigs, compared with the juveniles (12.10%). Some previous studies have also observed a higher seroprevalence of Aujeszky disease virus (ADV) between adults and juveniles [24]. However, non-significant difference (*P* > 0.05) was observed in *PR* seroprevalence between sex and feed type Tibetan pigs, which was different than the previous report [24]. Our results suggested that the incidence of PR was not related to the gender and feed type and there are several other possible reasons for this in Tibetan pigs. Firstly, it may be due to living in a similar environment and free-ranging system and secondly, each pig has the same chance to contact PRV [11, 25].

Hu et al. reported that the probability of reintroduction of PRV infection in large-scale Tibetan pig farms was positively different with free-ranging Tibetan pig farms [22]. However, our study has shown that feed type is associated with the infection of PRV. The subsequent univariate analysis indicated that the large-scale Tibetan pig farms were at higher risk for PRV seropositivity than free-ranging Tibetan pigs (OR: 1.11). Tibetan pigs are mainly fed by grazing in open environment [26]. Under such conditions, this animal gets more chance to expose to the external environment. Among these, sub-adults and adults have the longest time of outdoor activities which increases the risk of infection as compared with the juvenile Tibetan pigs.

Tibet is located in the Himalaya region and have high altitude with low temperature [10, 12, 26]. The local herdsmen have very less chance of interacting with the outside, which is not conducive to the survival of PRV. Tibet has unique mountains and rivers, forming a natural barrier structure, which is a different than reported earlier [27]. However, our study found that Tibetan pigs have been exposed to PRV and we discovered that the pigs living at low altitude (2800 m) were at higher risk for PRV seropositivity than those living at high altitude (3200 m and 3700 m). Current study reported PRV in Tibetan pig, and thus preventive and hygienic measures are suggested to minimize the serious economic losses. The vaccination, regular disinfection, and isolation of infected pigs are important to prevent the spread of PR from a particular area.

## Conclusions

The present study demonstrated the seroprevalence of PRV infection in Tibetan pigs, and found that feed type, age and altitude are major risk factors associated with PRV herd seropositivity and possible source for the spread of PRV in Tibetan pigs.

## Abbreviations

ELISA: Enzyme-linked immunosorbent assay
PR: Pseudorabies
PRV: Pseudorabies virus
SAS: Statistical Analysis System
